# Monitoring individual tree‐based change with airborne lidar

**DOI:** 10.1002/ece3.4075

**Published:** 2018-04-24

**Authors:** Laura Duncanson, Ralph Dubayah

**Affiliations:** ^1^ NASA Goddard Space Flight Center Biospheric Sciences Lab Greenbelt Maryland; ^2^ Department of Geographical Sciences University of Maryland, College Park Maryland

**Keywords:** airborne lidar, biomass, change detection, forest carbon monitoring, individual tree change, tree growth, tree mortality

## Abstract

Understanding the carbon flux of forests is critical for constraining the global carbon cycle and managing forests to mitigate climate change. Monitoring forest growth and mortality rates is critical to this effort, but has been limited in the past, with estimates relying primarily on field surveys. Advances in remote sensing enable the potential to monitor tree growth and mortality across landscapes. This work presents an approach to measure tree growth and loss using multidate lidar campaigns in a high‐biomass forest in California, USA. Individual tree crowns were delineated in 2008 and again in 2013 using a 3D crown segmentation algorithm, with derived heights and crown radii extracted and used to estimate individual tree aboveground biomass. Tree growth, loss, and aboveground biomass were analyzed with respect to tree height and crown radius. Both tree growth and loss rates decrease with increasing tree height, following the expectation that trees slow in growth rate as they age. Additionally, our aboveground biomass analysis suggests that, while the system is a net source of aboveground carbon, these carbon dynamics are governed by size class with the largest sources coming from the loss of a relatively small number of large individuals. This study demonstrates that monitoring individual tree‐based growth and loss can be conducted with multidate airborne lidar, but these methods remain relatively immature. Disparities between lidar acquisitions were particularly difficult to overcome and decreased the sample of trees analyzed for growth rate in this study to 21% of the full number of delineated crowns. However, this study illuminates the potential of airborne remote sensing for ecologically meaningful forest monitoring at an individual tree level. As methods continue to improve, airborne multidate lidar will enable a richer understanding of the drivers of tree growth, loss, and aboveground carbon flux.

## 1 INTRODUCTION

The terrestrial carbon sink is ~3.0 Gt/year (Le Quéré et al., 2015), much of which is driven by forest disturbance and regrowth. Tree growth and mortality are the primary drivers of aboveground carbon flux in forests (Houghton, 2005). Our current understanding of tree growth and mortality is primarily derived from field datasets, although changes in system productivity have been well documented through repeat observations of passive optical instruments such as Landsat and MODIS (Hansen et al., 2008; Huang et al., 2009; Jin & Sader, 2005). Additionally, repeat‐pass lidar, either from airborne platforms (Dubayah et al., 2010; Hudak et al., 2012) or spaceborne platforms (Dolan et al., 2011), has provided estimates of pixel‐ or plot‐level biomass change. However, none of these remote sensing‐based change analyses are capable of capturing detail at an individual tree level and rely on the aggregation of reflectance at a 30 m or 1 km resolution. Given that aboveground biomass flux in forests is primarily a function of the balance between tree growth (sequestration) and mortality, monitoring change at an individual tree level will enable an ecologically meaningful understanding of forest biomass dynamics.

Tree growth and mortality are not only of interest for monitoring the carbon dynamics of a forest, but also increasing our understanding of the limitations of growth and drivers of mortality. With respect to understanding tree growth, there remains debate regarding the biophysical drivers that prevent trees from reaching heights much greater than ~130 m (Koch, Sillett, Jennings, & Davis, 2004). Studies have predicted the maximum potential height as a function of environment (Shi et al., 2013) but it remains unknown when, if ever, trees will reach these potential heights. Additionally, the mechanistic constraints governing tree height are uncertain and remain an area of ecological interest (Domec et al., 2008). Additionally, our understanding of the drivers of tree mortality remains limited. In the United States, it has been documented that mortality rates are increasing (Van Mantgem et al., 2009), likely related to warmer temperatures and increases in drought, fire, and pathogens (Allen et al., 2010). Understanding the processes driving tree mortality, and monitoring mortality over time, is essential to modeling forest responses to climate change.

Existing methods for monitoring tree growth and mortality are generally focused on sparse, relatively small forest plot measurements. Tree growth is typically monitored based on measuring changes in stem diameter over time in field plots (Clark, Piper, Keeling, & Clark, 2003; Sillett et al., 2010) or by studying tree‐ring records (Briffa et al., 1998; Vaganov et al., 1999). Stem diameters cannot be systematically measured from airborne or spaceborne systems, limiting the spatial scale over which research can be conducted. Similarly, tree mortality rates are monitored by identifying tree death in monitoring plots. Remote sensing provides the opportunity to monitor forest dynamics from airborne and spaceborne platforms (Bolton, Coops, & Wulder, 2015; Hansen et al., 2008; Yu, Hyyppä, Kaartinen, & Maltamo, 2004) and has revolutionized our understanding of the carbon balance in Earth's forests.

To monitor individual tree growth and mortality, remote sensing data sensitive to individual tree scales are required. Airborne lidar instruments have been demonstrated as capable of capturing individual tree‐level detail across landscapes (Duncanson, Cook, Hurtt, & Dubayah, 2014; Morsdorf et al., 2004; Popescu, Wynne, & Nelson, 2003) and have been used to monitor pixel‐ or stand‐level change (Dubayah et al., 2010; Hopkinson, Chasmer, & Hall, 2008; Huang et al., 2013; Vepakomma, St‐Onge, & Kneeshaw, 2011). To date, several studies have demonstrated the ability to monitor individual tree‐level changes with multidate lidar (Asner & Levick, 2012; Levick & Asner, 2013; Levick, Baldeck, & Asner, 2015; Yu et al., 2004; Zhao et al., 2018). These studies focused on considerably shorter, lower biomass forests than those in our study, either in boreal systems (Yu et al., 2004; Zhao et al., 2018) or savannas (Levick and Asner), and therefore provided only limited insights into the relationships between tree height changes in tall forests approaching their theoretical height limits. We expand on the methods presented by Yu et al. (2004) and Zhao et al. (2018) to further explore the utility of multidate lidar for studying growth and mortality at an individual tree level in a tall, mature, high‐biomass system. The goals of this study were to quantify tree growth and removal at an individual level and assess the structural dynamics at Teakettle Experimental Forest with respect to changes in tree height and aboveground biomass.

## 2 MATERIALS AND METHODS

### 2.1 Study area

The study area, as outlined in Hyde et al. (2005), is an experimental forest in the Western Sierra Nevada Mountain range in California. Dominant species include *Abies concolor* (white fir), *Pinus ponderos* (ponderosa pine), *Abies magnifica* (red fir), and *Quercus kelloggii* (California black oak) (Honaker et al., 2002). The elevation range of the site is approximately 1,000–2,500 m above sea level, with aboveground biomass values averaging 200 Mg/ha with individual tree values up to 20 Mg per tree. The forest is mature, with rocky outcrops intermixed between clusters of trees. The primary disturbance affecting the broader ecosystem is fire, but no fire has occurred at Teakettle for over a century (North et al., 2004). Teakettle is in a National Forest, and thus, forestry activities are permitted and there is heavy logging in the area, particularly since 2000. Finally, California experienced a significant drought during this research period (2009–2015) (AghaKouchak, Cheng, Mazdiyasni, & Farahmand, 2014), which may have reduced tree growth rates (Dobbertin, 2005; Swatantran, Dubayah, Roberts, Hofton, & Blair, 2011). Indeed, Swatantran et al. (2011) found evidence of increased stress at Teakettle during this period through an analysis of AVIRIS data.

### 2.2 Field and lidar data

Twelve, 90‐m^2^ plots were established in the summer of 2008 across Teakettle Experimental Forest. Measurements of individual tree heights with vertex hypsometers, stem diameters, and crown radii were measured in each plot.

Lidar data were acquired over Teakettle in the summers of 2008 and 2013. The first lidar acquisition was flown using the University of Florida's Optech Gemini ALTM, operating at 100–125 kHz with a maximum 25 degree scanning angle, a wavelength of 1,064 nm, and a nominal footprint diameter of 15 cm. The average return density was approximately 18 returns/m^2^. Lidar data in 2013 were acquired by NASA Goddard's Lidar, Hyperspectral and Thermal Imager (G‐LiHT, Cook et al., 2013). G‐LiHT uses a 300 kHz multistop scanning lidar operating at 1,550 nm with a 60° field of view and 10 cm diameter footprint. The average return density was slightly lower than the first lidar acquisition, at approximately 13 returns/m^2^.

### 2.3 Lidar processing

Once discretized into point clouds, both lidar datasets were processed identically, using a crown delineation algorithm described in Duncanson et al. (2014). The algorithm performs a multistory delineation based on a modified watershed approach, which produces a Digital Elevation Model (DEM), smoothed canopy height model (CHM), and a preliminary delineation using a watershed algorithm similar to Chen, Baldocchi, Gong, and Kelly (2006). Each preliminary segment is subsequently refined using the lidar returns from within each segment. Returns are classified as either overstory or understory depending on their vertical location within a segment, and each set of returns (overstory and understory) is processed individually, through the generation of an overstory and understory CHM. These secondary CHMs are then segmented, and the process is iterated until no further understory layers are detected. This method therefore iteratively separates the local canopies into layers and delineates both overstory and understory trees through the generation of multilayers CHMs. At Teakettle, there were only two iterations of this process, producing one overstory and one understory set of delineated crowns. For each crown, the area, radius, height, and unique ID were extracted. The original algorithm extracted height as the maximum return height within each crown, but for this analysis, we instead extracted the maximum smoothed CHM value, in an attempt to minimize differences in maximum tree height caused by differences in lidar point density.

### 2.4 Lidar change detection

The Optech and G‐LiHT datasets differ not only in lidar wavelength, but also in return density, flight lines, side lap, scan angle, etc. These differences are potentially problematic for change detection. To minimize lidar discrepancies, data from each year were filtered to a maximum scan angle of ±15° to reduce differences in crown delineations associated with wide scan angles, where one side of a crown may be occluded from the sensor. We assume that, in the resulting areas of overlap, given the high return densities of both datasets (~13–18 returns/m^2^) and the open structure of the forest at Teakettle, both datasets provide sufficient sampling density to produce an accurate DEM, above which CHMs and individual crown delineations can be compared. There was an apparent spatial mismatch between many of the delineated crowns in 2008 and subsequent delineations in 2013. Differing flight lines, scan angles, and flight conditions (pitch, roll) yielded slight differences in the location of tree crowns, and these spatial mismatches were not consistent across the study area. However, the open canopy conditions of the forest allowed easy manual identification of dominant trees between the years. Image‐to‐image registration was applied using 50 ground control points to remove the apparent spatial mismatches.

### 2.5 Tree growth

Each tree crown was automatically assigned an identification number as part of the watershed delineation and extracted crown dimensions are associated with these numbers. To match trees in 2008 to trees in 2013, individual tree crown centroid locations from 2008 were overlain on top of the geo‐registered raster of delineated 2013 crowns. The 2013 IDs were extracted and used to match the attributes of crowns between the 2 years. This method can be problematic if crown delineations differ between the years (e.g., a single crown may be over‐ or undersegmented in 1 year due to a difference in lidar point density). To ensure our growth analysis was based only on trees that were properly matched between the 2 years, we filtered data to include (1) only trees where the crown centroid from 1 year was within the delineated canopy from the other year, (2) only trees with less than 5 m change in height, (3) only trees that had less than a 1 m difference in underlying DEM elevation, and (4) only trees that had less than a 100% change in estimated crown radius (i.e., filtering any mortality events or partial crown segments from the growth analysis). Iterations of each of these filters were applied, and these filters were selected to maintain as large a sample size as possible while maintaining the shape and/or magnitude of growth results (Figures S2–S4). Changes in tree height were set to the difference between the maximum CHM value of a crown from 2013 minus the maximum CHM value from the same crown in 2008. The distribution of changes in tree height was analyzed as a function of tree height in 2008, where the distributions of changes in tree height were plotted against height class itself, thus allowing an analysis of the influence of tree size on growth rate.

### 2.6 Tree loss

**Figure 1:**
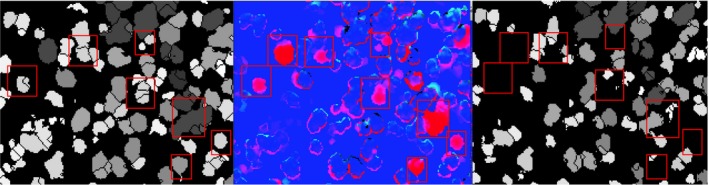
Tree loss detection is based on a watershed segmentation of the canopy height model (CHM) from 2013 to 2008. If a CHM change matches a delineated crown segment from 2008, a tree loss event is flagged. The detected tree losses above are depicted by the boxes

Our tree loss detection involved the development of a watershed‐based algorithm. First, we subtracted the 2013 CHM from the 2008 CHM (Figure 1), yielding a CHM difference map. We applied a watershed segmentation to this difference map and matched segments from the difference map to the 2008 CHM to identify crowns that were removed from the 2008 dataset. There were also several small segments delineated from the edges of crowns, presumably because the lower point density or smaller footprints in the 2013 dataset that were less sensitive to the edge of conifer crowns, and thus underestimated crown radius (Figure 4b). The algorithm searched for similarities in delineated crowns in the CHM difference delineation layer (tree loss layer) that matched crowns in the 2008 layer. A tree loss was detected when the 2008 and CHM difference (tree loss) segmentations had less than a 3 m difference in height and 30% difference in crown area. Details of the selection of these filters are provided in Figures S5–S8. As with tree growth, the mortality rate was analyzed as a function tree size.

### 2.7 Biomass change

**Figure 2:**
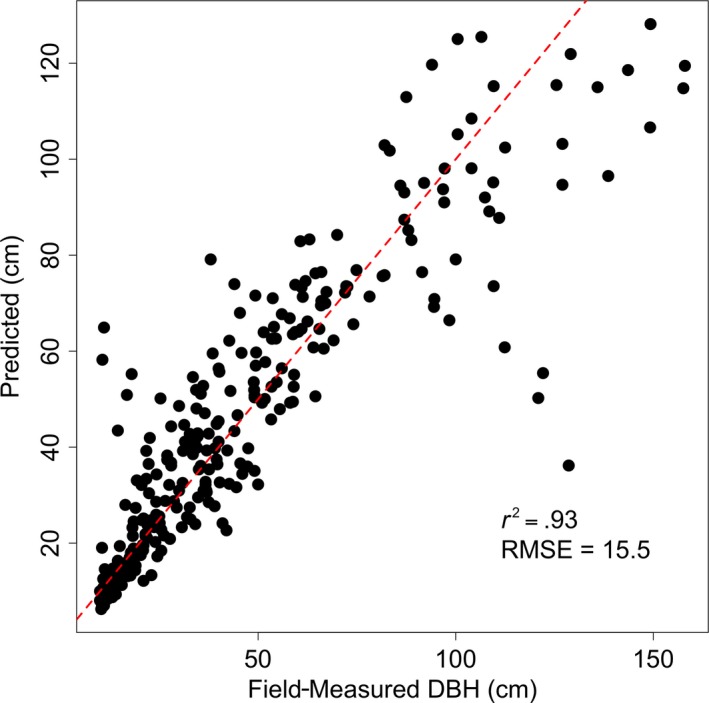
A relationship was developed between field‐measured diameter at breast height (DBH) and field‐measured crown radius and height, where DBH = 2.2 × Ht ± 15.5, %RMSE = 21.6%. This relationship was then applied to estimate the DBH of every tree across the overlapping lidar area

We estimated the individual tree aboveground biomass for every tree delineated in the overlapping lidar acquisitions. We employed the Jenkins et al. (2003) generalized allometric equation for true fir/hemlock species group, as this best describes the majority of the tree species found at Teakettle, as follows:

(1) Biomass=exp[−2.5384+2.4814×ln(DBH)]

where biomass is expressed in kg per tree, and DBH is the diameter at breast height in centimeters. The reported standard error of this equation, in log units, is 0.182329. To use this equation, we require stem diameter data, which we do not have at the site level. However, we developed an empirical relationship predicting DBH as a linear function of individual tree height from our field dataset (Figure 2), similar to methods applied by Jucker et al. (2017). We applied this DBH model to the full lidar‐derived tree height dataset and applied Equation (1) to estimate the biomass per tree. We summed the biomass of all trees in the overlapping area of each acquisition year to estimate the biomass change between the two periods, as well as estimating the biomass change per size class.

### 2.8 Error propagation

The developed methodology relies on individual tree extraction from a multilayered crown delineation algorithm from each time period, which has associated errors in each crown extraction, as well as well as in the application of allometric equations both generated in this study (to height to stem diameter) and from the literature (to relate stem diameter to biomass). We propagated errors through each step of the analysis to determine the uncertainty of our final biomass change assessment, following popular error propagation methods for biomass (Ahmed, Siqueira, Hensley, & Bergen, 2013; Chave et al., 2004; Chen, Vaglio Laurin, & Valentini, 2015).

We assume negligible errors in our field measurements of stem diameter, height, and crown measurements. Although there is certainly some measurement error, we have no means by which to quantify it, as repeat field measurements are not available. Similarly, we do not have stem‐mapped crown measurements to quantify errors in delineated crown dimensions from the field. Instead, we assume that the tallest, most mature trees in our study will have negligible growth over between 2008 and 2013. We quantify errors in height estimates by comparing spatially matched individual tree crowns from 2008 to 2013 and calculating the mean and standard deviation (*SD*) of differences in trees >50 m in height. The *SD* of tall tree heights is used as an estimate of individual tree height measurement error. We assume this error is consistent across size classes as an absolute error, because accuracies in height do not improve for shorter trees. Errors in crown size extraction and height are associated with probability of detecting the apex of a crown or edge of a canopy, which will be sensitive to lidar point density and footprint size, but not to tree height.

This measurement error is propagated through our height to DBH model (Equation (2)), assuming independent and random errors associated with measurement error and error on the parameter fit on the scalar, *a*. The error associated with this model can be expressed as the root sum of squares of fractional errors (Equation (3));

(2) $$\text{DBH}=\left(a\times \text{ht}\right)\pm \sigma_{\mathrm{DBH}}$$

(3) $$\sigma_{\mathrm{DBH}}=\sqrt{\frac{\sigma_{a}^{2}}{a}+\frac{\sigma_{\mathrm{ht}}^{2}}{\text{ht}}}$$

where σ_DBH_ is the standard error on height‐derived estimates of DBH, σ_*a*_ is the standard error on the parameter fit on Equation (2), and σ_ht_ is the measurement error in crown‐delineated heights.

(4) $$\sigma_{\mathrm{ht}}=\text{mean}\left[\text{abs}\left({\text{ht}}_{2013>50}-{\text{ht}}_{2008>50}\right)\right]$$

The DBH estimates per tree are then used to estimate individual tree biomass through Equation (1) (Jenkins et al., 2003). Error in our estimates of DBH is included in this model following Equations (5) and (6),

(5) $$\frac{\sigma_{m\_\mathrm{dbh}}^{2}}{M}=\frac{\sigma_{\mathrm{DBH}}^{2}}{{\mathrm{DBH}}^{2}}{\left(\frac{\partial \ln(f)}{\partial \ln(\mathrm{DBH})}\right)}^{2}$$

(6) $$\sigma_{m\_\mathrm{dbh}}=M\times 2.4814\frac{\sigma_{\mathrm{DBH}}}{\text{DBH}}$$

where σ_DBH_ is our estimated uncertainty from our DBH model (including height measurement error). Equations (1) and (5) are combined to estimate the error in biomass with respect to DBH error, σ_*m*_dbh_, as the product of the estimated biomass, *M*, the allometric exponent, and the fractional error in DBH. This is combined as a sum of squares with the estimated parameter error on the allometric exponent, following (Ahmed et al., 2013),

(7) $$\sigma_{\mathrm{AGB}}=\sqrt{\sigma_{m\_{\mathrm{dbh}}^{2}}+\sigma_{a}^{2}}$$

(8) $$\sigma_{a}=\text{exp}\left(2\sigma^{2}+2\mu \right)-\text{exp}\left(\sigma^{2}+2\mu \right)$$

where μ is the log of estimated biomass, and σ is the reported error in log space, in this case 0.18.

The total biomass across the study site will have an error associated with the sum of individual tree errors, while the total change in biomass will have an error of twice the percentage error of the total, Equation (10).

(9) $${\text{AGB}}_{\mathrm{change}}=\sum_{i}^{n}{\text{AGB}}_{i\_2013}-\sum_{i}^{n}{\text{AGB}}_{i\_2008}$$

(10) $$\sigma_{\mathrm{change}}=\frac{\sum_{i}^{n}\sqrt{\sigma_{\mathrm{AGB}\_i\_2013^{\sigma }}}}{\sum_{i}^{n}{\text{AGB}}_{i\_2013}}+\frac{\sum_{i}^{n}\sqrt{\sigma_{\mathrm{AGB}\_i\_2008^{\sigma }}}}{\sum_{i}^{n}{\text{AGB}}_{i\_2008}}$$

## 3 RESULTS

**Figure 3:**
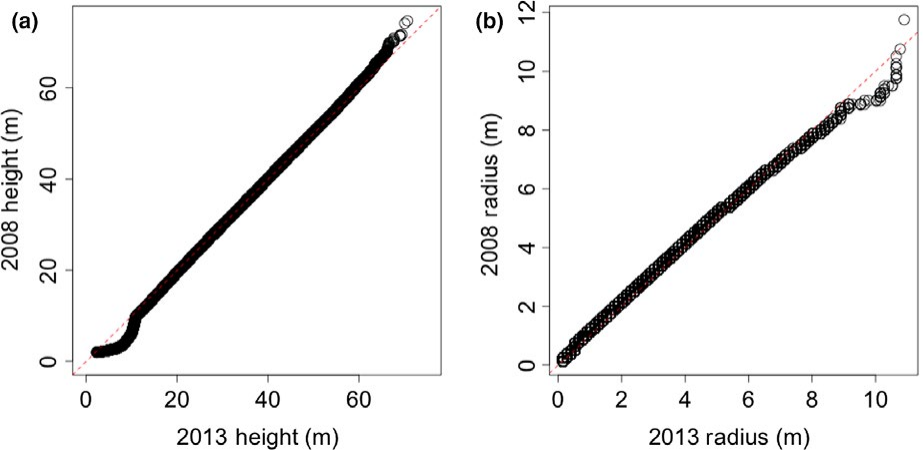
The distributions of height and crown radius between 2008 and 2013 fall along the 1:1 line in a quantile–quantile plot (a), while there is a slight underestimation of crown radius found in 2013 (b), likely because of a lower point density

**Figure 4:**
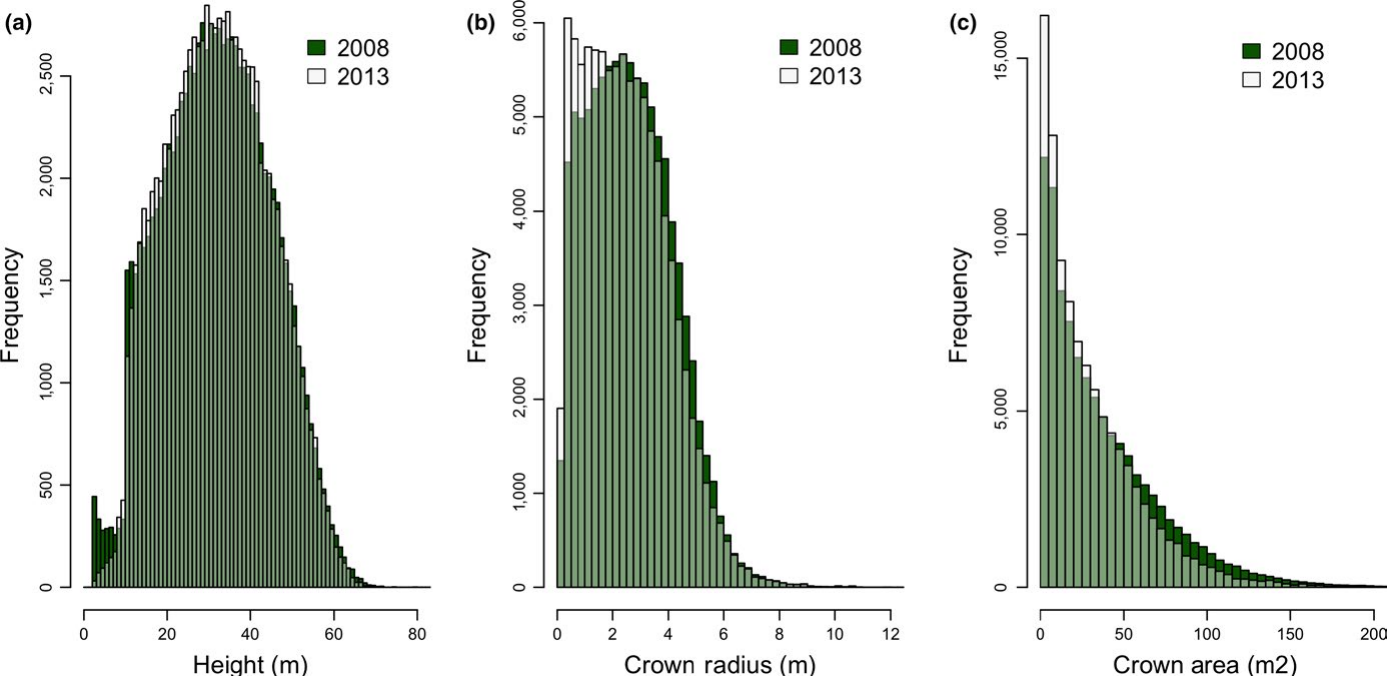
Histograms from 2008 to 2013 of tree height (a), crown radius (b), and crown area (c)

There were 87,913 crowns delineated in 2008 and 79,078 delineated in 2013 in the study region with overlapping lidar with less than 15° scan angle. Distributional quantile plots (Figure 3) comparing canopy heights and crown radii show that the general patterns in tree heights and crown radii are preserved between the 2 years. However, histograms comparing the two distributions show a systematic bias in crown radius, with 2013 extracting relatively smaller radii than 2008 (Figure 4). While the two datasets yielded similar distributions of crown attributes, the height estimates appear unbiased except for trees >60 m (Figures 3a and 4a). The G‐LiHT data not only had a lower nominal point density, but also had less overlap and a smaller nominal footprint than the 2008 dataset. These differences likely explain the observed bias in crown radius, with smaller extracted crowns in 2013 (Figure 4b). We therefore focus on an analysis of growth increase with respect to height, rather than a change in crown dimensions.

### 3.1 Tree growth

The mean pixel values of the CHM from 2008 and 2013 were 10.18 and 11.73 m, respectively, suggesting an average increase in forest height of 1.55 m. However, this value is the average change in total height, not individual tree height, and does not take into account trees that were lost during between 2008 and 2013. The mean tree heights in 2008 and 2013 were 31.54 and 32.05 m, respectively. This suggests an average growth rate of 0.5 m over the 5‐year period or 10 cm per year. Because of spatial mismatches and slight differences in segmented crowns caused by differences in the lidar acquisitions, we filtered the sample of trees used for growth analysis to only include trees that were delineated in both years with similar heights and crown dimensions (to ensure we do not compare changes of neighboring or partial crowns). The resulting sample size after filtering to include only matched trees between the 2 years resulted in a sample size 29,247 trees.

**Figure 5:**
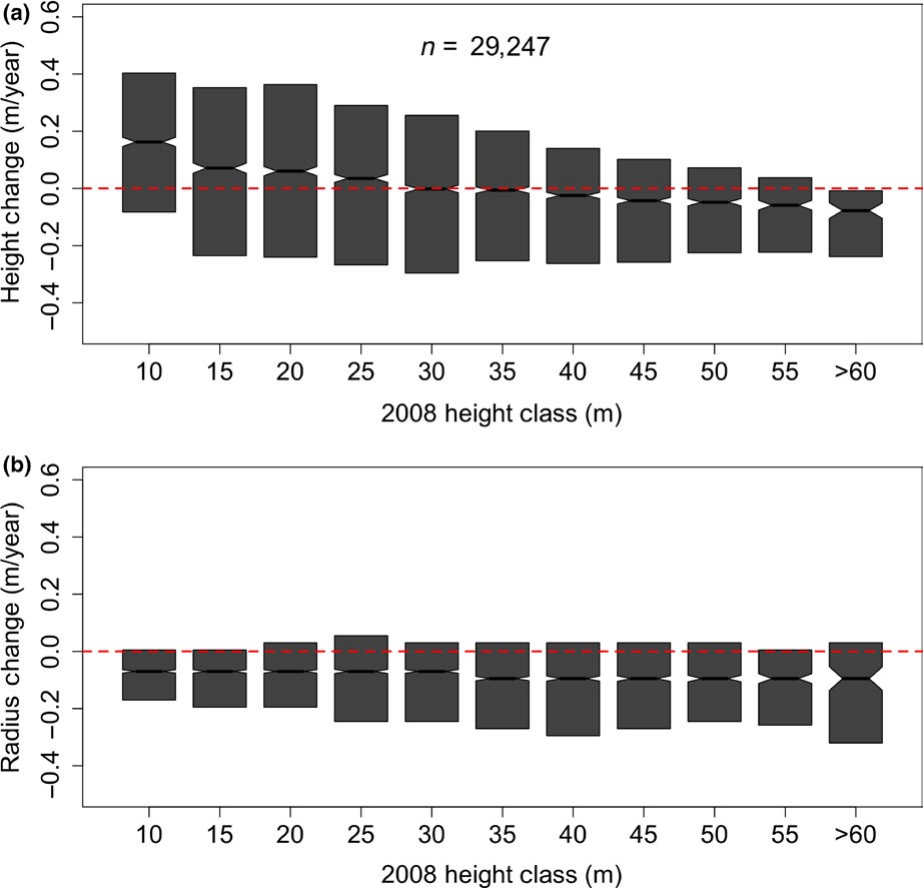
Growth rate in height (a) consistently decreases with increasing tree size, while the distribution of rates in each size class remains relatively consistent. The gray bars represent the 10th to 90th percentile distributions for each height class. Notches represent an approximate 95% confidence interval for the median. Growth rate in radius (b) was consistent and slightly negative. This confirms that crown radii were either being overestimated in 2008 or underestimated in 2013. Despite this bias in radius delineation, there is no apparent change in crown radius change as a function of tree size. This suggests that crown size may not impact radial expansion of crowns at Teakettle

Delineating trees allows the analysis of not only average changes in height, but also changes in height as a function of tree size (height and radius). The median, maximum, and minimum growth rate per size class all decrease with increasing tree height (Figure 5a).

We also assessed changes in crown radius with respect to tree size (Figure 5b), which showed that, while the overall change was negative (i.e., the extracted crown sizes were smaller in 2013 presumably due to differences in lidar acquisitions), there was no appreciable difference in crown change with respect to size. This suggests that any lateral growth in tree crowns was consistent across size classes.

Observed negative growth rates in height and crown radius are attributed to lidar acquisition differences, where the lower nominal point density and smaller footprint size of the 2013 campaign reduced the probability of the 2013 data to yield returns at the apex and outer edges of tree crowns. This illuminates the sensitivity of the crown delineation approaches here to lidar specifications.

### 3.2 Tree loss

**Figure 6:**
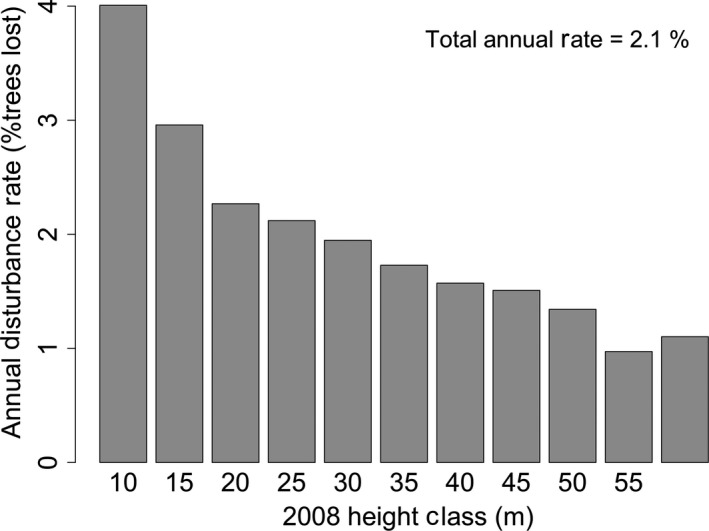
The disturbance or tree loss rate in the system decreases as a function of tree size. These rates are for the full 5‐year period; thus, the overall annual tree removal rate is 2.1%

The site‐level delineation result yields a difference of 8,835 trees, suggesting a 10% loss in trees over the 5‐year period. This loss is from comparing histograms rather than identifying individual mortality events. The watershed‐based tree loss detection yielded an estimate of 9,199 trees removed from the landscape or 10.4% (1.84% annually). The similarity of these two numbers suggests that our watershed‐based loss identification approach yields reasonable site‐level estimates of tree loss. Figure 6 shows the loss rate (number of trees removed from landscape divided by total number of trees) as a function of tree height class. The disturbance rate decreases with increasing tree size, suggesting that shorter trees, at Teakettle, are more susceptible to disturbance.

### 3.3 Biomass change

**Figure 7:**
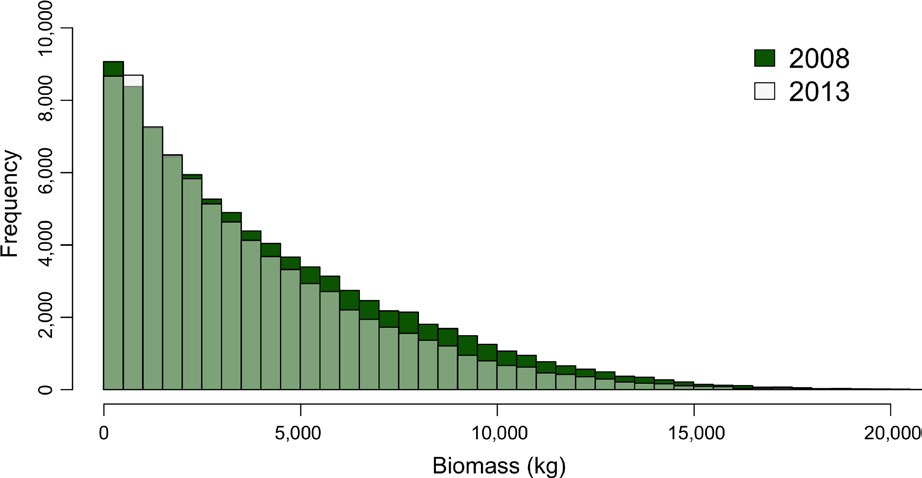
Histograms comparing the estimated distribution of individual tree biomass in 2008 and 2013 show that the tree size distribution remains consistent between the years, but biomass losses are greater in higher biomass strata, suggesting that large tree loss dominates the aboveground carbon dynamics of this system

To estimate the change in biomass, we included a propagation of errors from both allometric models and individual tree height estimates. The total estimated biomass in 2008 was 359.67 ± 208.53 Gt (58%), which was reduced to 285.09 ± 172.73 (60%) Gt in 2013 (Figure 7). The total loss in estimated aboveground biomass was therefore 74.578 ± 62.54 (83%) Gt, approximately 20% ± 16.6% of the total biomass, or ranging from a loss of 4% biomass to a loss of 36% biomass. These large errors are attributed to the high per‐tree errors, both in the DBH estimates and biomass model. The DBH estimates had an average error of 24%, incorporating estimated measurement error in tree height (7.7%), DBH model parameter error (6.3%), and DBH residual error (21.7%). These DBH errors were amplified through the application of the Jenkins nonlinear biomass equation, where 24% error in DBH yielded a nominal 61% error in per‐tree biomass. This per‐tree biomass error is attributed to ~18% in allometric fit error, as reported by Jenkins et al. (2003), and ~58% error from propagation of DBH errors through the exponential biomass prediction model.

**Figure 8:**
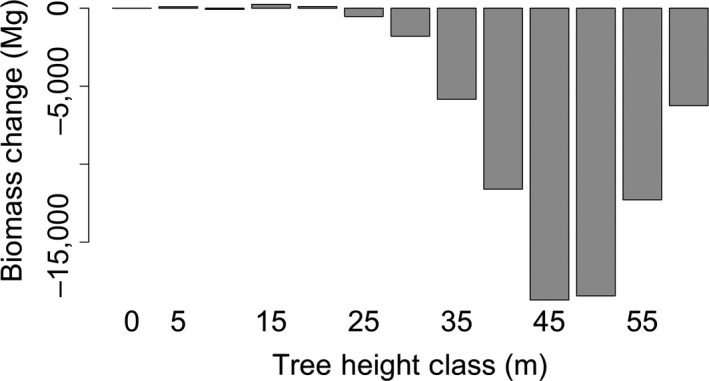
The change in biomass at Teakettle shows that Teakettle is an overall carbon source (loss of ~74 Gt of carbon), with most aboveground biomass being lost large trees (>30 m)

To determine which size classes predominantly lost biomass, we plotted changes in biomass with respect to tree height classes in 2008. Most carbon is being lost from large trees (>30 m in height), where growth is minimal. Smaller trees (<25 m) have the highest growth rates (Figure 5) but also the highest removal rates (Figure 6), and therefore, these size classes are essentially carbon neutral with respect to aboveground biomass (Figure 8). However, considering the decrease in height for the tallest trees in the system (Figure 5a), the loss of biomass for the largest individuals may be partially explained by biases in height between the two lidar acquisitions.

## 4 DISCUSSION

In this study, we implement an approach for analyzing forest change at an individual tree level from multidate lidar. Our methods for tree growth rely on comparing spatially matched individual crowns from two lidar acquisitions with different instruments, flight specifications, point densities, etc. Although the digital elevation models were consistently extracted between the years, differences in the acquisitions yielded differences in segmentation products, including local spatial discrepancies, partial crown segmentations, and nominally smaller extracted crowns from the second acquisition. This work illustrates that individual tree analysis from multidate lidar is challenging, and careful attention to lidar sampling design is recommended. Despite the challenges of individual tree monitoring with lidar, we demonstrate that trends in individual tree growth and loss can be assessed at a landscape scale with sample sizes orders of magnitude larger than typically collected by a single project in the field.

### 4.1 Tree growth

We show that tree growth rates vary as a function of tree height. Short trees (~10 m) in this study area are growing ~80 cm over the 5‐year period, with an average annual growth of ~13 cm/year. This growth rate is within the range observed by the Forest Inventory Analysis for the Fresno County (Figure S1). As trees increase in height, we see a decrease in growth rates, as expected, with the tallest trees (>60 m) showing slightly negative growth rates (~ −10 cm/year). This is either due to physical damage to treetops in response to a driver such as drought or wind damage or due to an underestimation of height in the second lidar acquisition. We expect trees to be slow growing in this system, particularly due to the California drought during the study time period, which should retard increases in height as trees approach their theoretical height maxima. Considering the apparent decrease in height for the tallest trees in the system, which we assume is related to lidar errors rather than ecological phenomena and that growth in height for trees >60 m is negligible (Koch et al., 2004; Ryan & Yoder, 1997), we focus on the trend in height change rather than the absolute magnitude of the change. Even if there is a bias between the heights extracted by the two datasets, there remains a clear trend in decreasing growth rates with tree height. We did not observe a trend between lateral growth rate and tree size, suggesting that, in this system, crown expansion may not depend on tree height. We emphasize that multidate lidar monitoring of forest growth is sensitive to instrument and flight characteristics, and therefore, change detection from multidate lidar should focus on data acquired in a consistent fashion.

### 4.2 Tree loss

Monitoring tree loss with multidate lidar is simpler than assessing individual tree change because the magnitude of the change in structure is much larger. Our tree loss algorithm will be sensitive to both natural and anthropogenic tree removals from the landscape. Our lidar‐based change detection will not resolve pathogen, drought, or insect‐driven mortality unless these mechanisms have resulted in a tree falling over or being removed, and thus, this study does not present a methodology for tree mortality monitoring, but only tree fall.

The mortality at Teakettle may not reflect typical old‐growth conifer forests, as the site has a history of fire suppression and has had increased logging since 2000 (North et al., 2002). The primary natural drivers of mortality are bark beetle, root disease, and dwarf mistletoe, working in tandem with abiotic drivers such as drought. Despite decades of fire suppression, the system retains gaps associated with fire disturbance that have not been recolonized (North et al., 2002). Although these natural biotic and abiotic stressors may have a higher mortality rate in larger trees, as has been documented by field‐based studies (Smith, Rizzo, & North, 2005), our results show the opposite pattern with higher tree fall (or removal) rates in smaller trees. Our findings agree with other studies that have demonstrated higher mortality rates in small‐ and medium‐sized conifers when compared with large trees (Van Mantgem et al., 2009). We observed 5‐year mortality rates of ~20% for the shortest trees analyzed (5–10 m) which decreased to the lowest rates of ~5% for the tallest trees (>60 m). The annual mortality rates, then, are 4% for the shortest trees and 1% for the tallest trees, with an average system‐level mortality rate of 2.01%/year, generally consistent with field observations in the Western United States (Van Mantgem et al., 2009).

### 4.3 Carbon balance

The estimated carbon loss from the system (~20%) reflects both changes in individual tree growth and loss. At Teakettle, both growth and loss rates were highest for the shortest trees, yielding near carbon neutrality for trees ~25 m. Larger trees had lower growth rates (assumed negligible after 50 m) and represented a net loss of biomass, with peak losses from trees between 40 and 55 m of height. However, both our biomass stock and change estimates have high associated errors. Individual tree estimates of biomass had an associated error of ~60%, and this error was additive to the landscape level because we have focused on biomass totals rather than averages. The estimated loss of biomass (~20%) has an associated standard error of ~80%. The majority of this error is from error in the tree height‐estimated DBH (including measurement and modeling errors), which is amplified through the application of an exponential allometric equation. These errors still do not fully account for errors associated with the fitting of the adopted biomass allometric equation, which are likely underestimated especially for the larger trees in this study. If a study is focused solely on quantifying system‐level biomass change, or changes in regional height distributions, alternative pixel‐based approaches may be more appropriate than the individual tree approaches analyzed here (e.g., Dubayah et al., 2010; Huang et al., 2013; Kellner, Clark, & Hubbell, 2009). These approaches do not rely on crown extractions but on precise lidar‐based estimates of height, thus avoiding errors associated with crown extractions. Additionally, pixel‐based change is significantly less computationally demanding than the methods examined in this work. However, pixel‐based approaches do not provide information at the organism level and are often focused on the upper canopy, while growth may be dominant in young, understory, or mid story trees. Researchers should carefully consider whether they require individual tree‐level information prior to applying the methods explored in this manuscript for biomass monitoring.

## 5 CONCLUSIONS

Advances in lidar remote sensing have enabled the systematic extraction of individual tree information across landscapes, providing an unprecedented opportunity to conduct individual tree‐based ecology with much higher sample sizes across environmental gradients. In this study, we outline an approach to quantify tree growth, in terms of height, as well as tree fall/removal. The methods applied were both computationally demanding (we used high‐end computing to extract individual crowns) and not fully automated (requiring image‐to‐image registration) and, therefore, require evolution for automated application to much larger areas. Our results suggest that an individual tree approach is not only possible with multidate lidar, but can provide insights into organism‐level forest dynamics and carbon balance that are not possible at a pixel level. The greatest technical challenge of this work was matching individual tree crowns between the 2 years, as differences in the lidar acquisitions produced discrepancies in the size and location of delineated crowns. To overcome these issues, we filtered our data to limit our individual tree‐level analysis of growth to only include trees that were consistently delineated between the 2 years. We recommend that multidate Lidar studies use consistently acquired lidar datasets, which should reduce biases and spatial mismatches between lidar datasets. The watershed‐based mortality detection method enables the identification of tree removal events and is less sensitive to slight spatial mismatches between the acquisitions than our growth analysis.

We expect lidar‐based individual tree change will become increasingly popular as crown delineation algorithms continue to improve in both accuracy and computational efficiency. The methods explored in this study represent early analyses of individual tree change at a landscape scale, and considering the increasing availability of high point density lidar, we anticipate the extension of these methods to other forest systems and/or other crown extraction approaches in the near future. As such, we reiterate the importance of careful consideration of lidar specifications prior to conducting individual tree‐based change analysis.

We demonstrated that the growth and loss at Teakettle both decrease with increasing tree height. The growth analysis is unsurprising, as other studies have demonstrated decreases in height growth as trees age and approach their mechanical limits of tree growth. However, our finding of slightly negative growth rates for tall trees could either be a real ecological phenomenon (related to thinning at the top of the tree from stress, for example, Swatantran et al., 2011) or an artifact of differences in lidar acquisition specifications. If the latter, it is possible that our tree growth analysis is biased and the tree growth rates are higher than those reported in this work. This will not, however, affect the observed trend of decreasing growth rates for taller trees.

We also speculate that tree loss is primarily driven by harvesting in the area, with higher harvest rates for shorter trees. From a carbon balance perspective, the study area is a carbon source, losing ~20% (±16%) of its AGB over the 5‐year period (~4 ± 3.2%/year). The carbon loss in this system is primarily driven by the tallest size classes, where growth is slow and any tree loss contributes a significant loss of biomass. This underpins the importance of large trees in carbon dynamics in mature, conifer systems.

## CONFLICT OF INTEREST

None declared.

## AUTHOR CONTRIBUTIONS

LD designed the study, conducted the research, and led the writing of the manuscript. RD helped write the manuscript and provided analytical and statistical insights that significantly influenced the direction of the manuscript.

## DATA ACCESSIBILITY

The derived individual crown attributes used from this study will be made available on the Oak Ridge DAAC in the coming months.
