# Supplementary material for: Monitoring individual tree‐based change with airborne lidar
Source: Ecol Evol. 2018 Apr 24;8(10):5079–89. doi: 10.1002/ece3.4075 (PMC5980410; doi:10.1002/ece3.4075)
Supplement: Supplementary file 1 [file ECE3-8-5079-s001.docx]

Supplementary Information


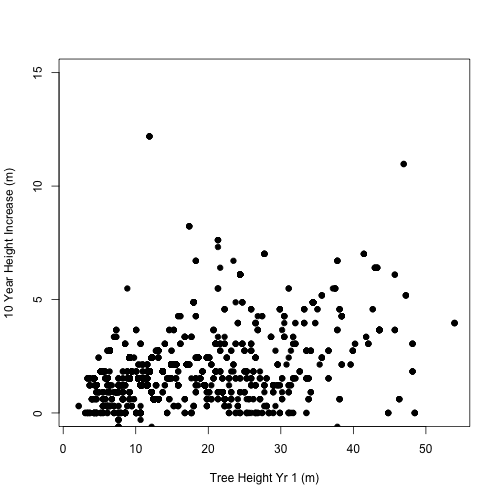


SI Fig 1. The U.S. Forest Service’s Forest Inventory Analysis (FIA) dataset was mined to find trees that were measured multiple times in the Fresno County. Trees that were measured with ~10 years between observations were included in this analysis. Although height measurements in the field are known to include significant errors, the range of 10 year height increases for short trees ranged to ~13 m increase. The increases of height found in this study are within the range of field observed changes in the County.


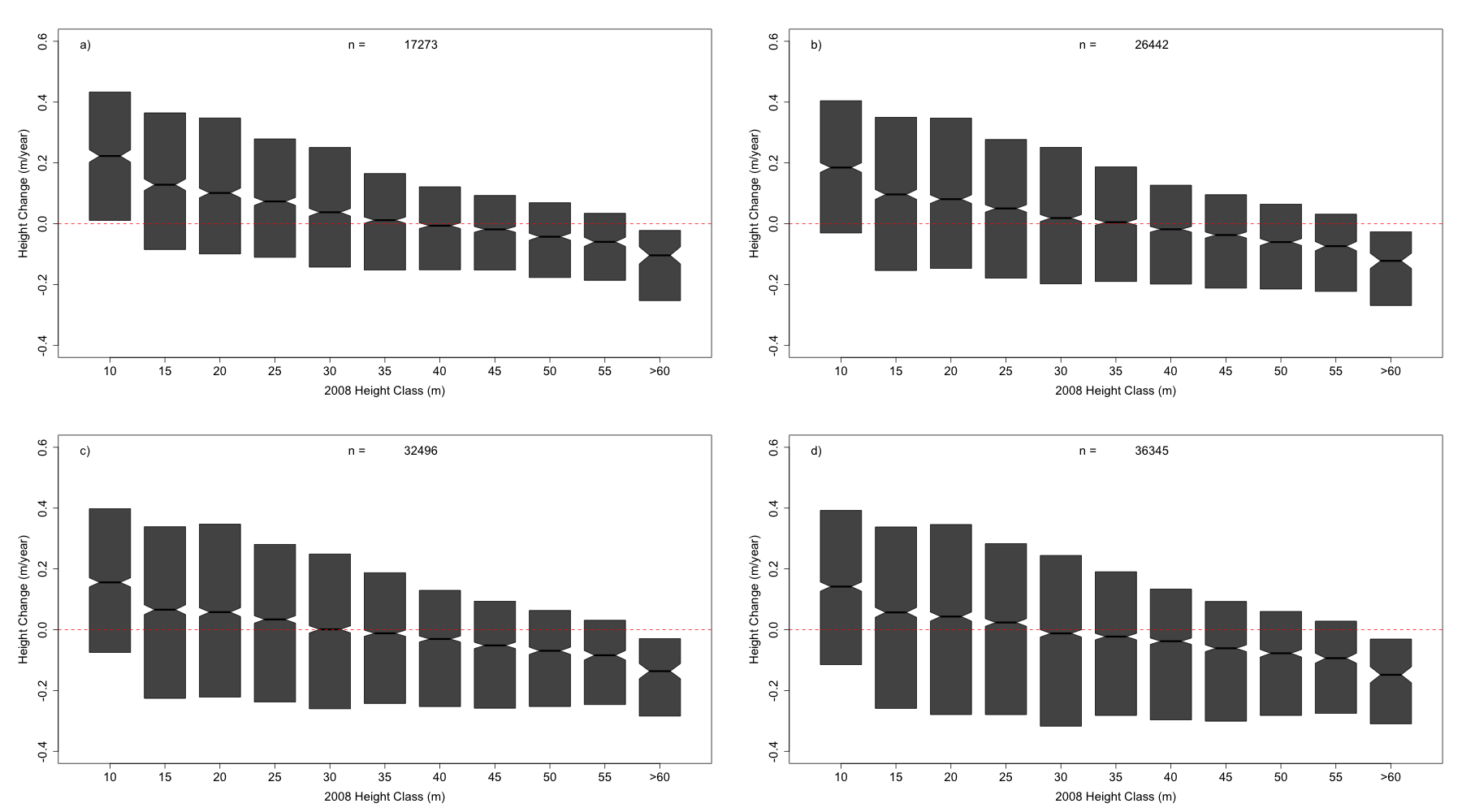


SI Fig 2. Applying different filters to the tree growth data to ensure the tree from year 1 were matched to the trees in year 2. These are the changes from changing the percentage of radius filters. A) shows the results filtering for only delineated crowns that differ in <25% crown radius, b) <50%, c) <75% and d) <100%


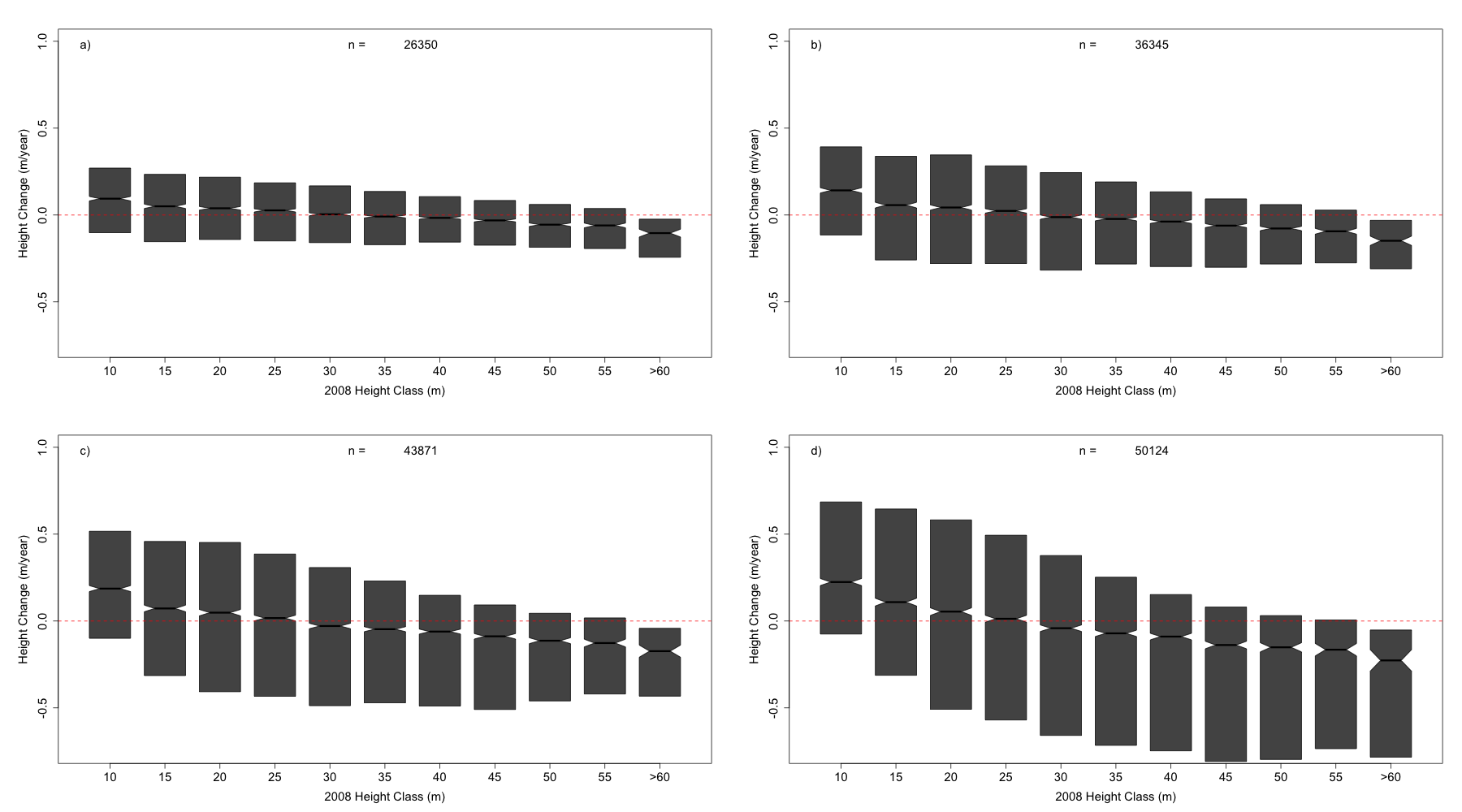


SI Fig 3. Showing the results in growth rate differing by filtering for different heights, where a) are heights <2.5 m different, b) heights <5 m different, c) <7.5 m and d) <10.0 m different


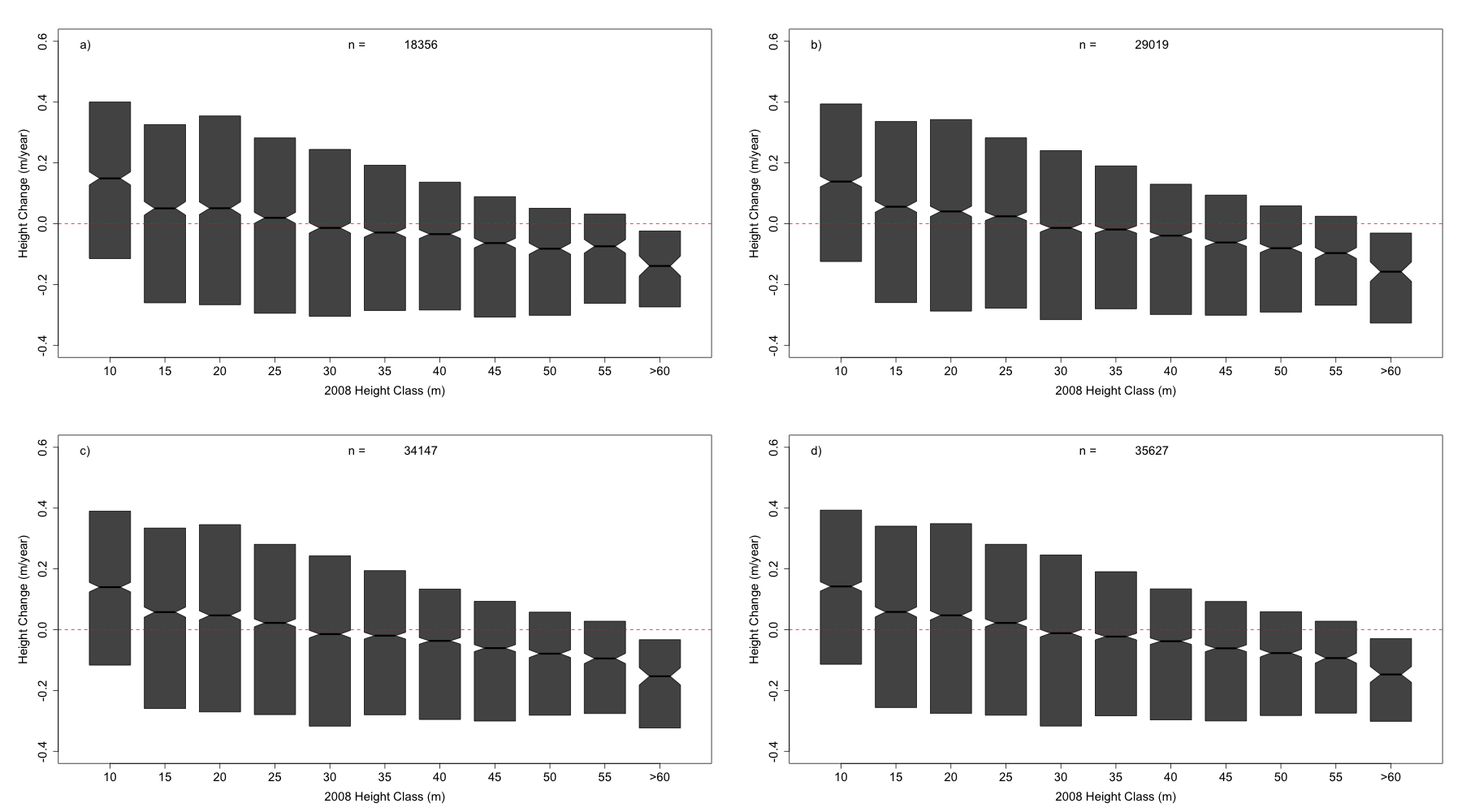


SI Fig 4. Showing the results in growth rate differing by filtering for differences in elevation derived under the canopy for the two years, where a) are elevation differences of <0.5 m different, b) <1.0 m different, c) <2.0 m and d) <5.0 m.

Also performed the filtering iterations for mortality. The watershed delineations from the CHM difference raster were matched to the original delineations from the 2008 lidar data. There were three filters applied to this dataset to perform the matching: 1) A distance filter, searching for delineations within a certain percentage of the radius of the 2008 delineated crown, 2) an area filter, filtering so that only segments within a certain percentage of the original delineated segment were kept and 3) a height filter, ensuring only segments within a certain height difference range were kept. Each of these three filters was iterated. We tested the radius distance filter, looking for segments whose centroids were within 1, 2, 3 and 4 times the radius of the original crown centroid. We also iterated over the acceptable area filter, keeping only delineations within 25%, 50%, 75% and 100% different from the original crown area. Lastly, we filtered for height, keeping only delineations within 2.0, 3.0, 4.0 and 5.0 m different from the original crown height. When iterating for any one of the filters we adopted the most conservative of the other two filters.


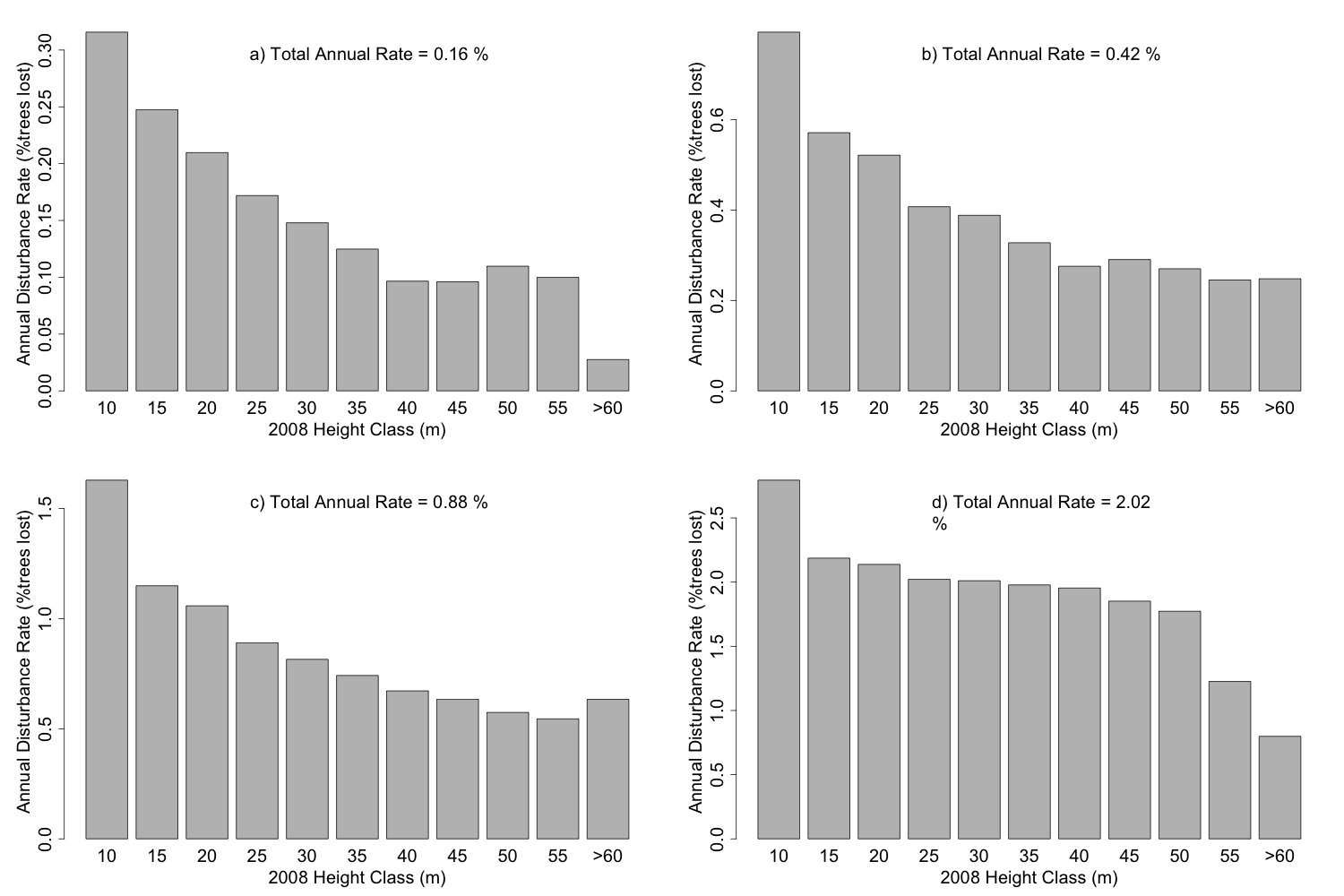


SI Fig 5. Classifies a tree loss only when the delineated loss is within 25% of the crown area of the original segmentation, b) classifies a loss within 50%, c) 75% and d) 100%


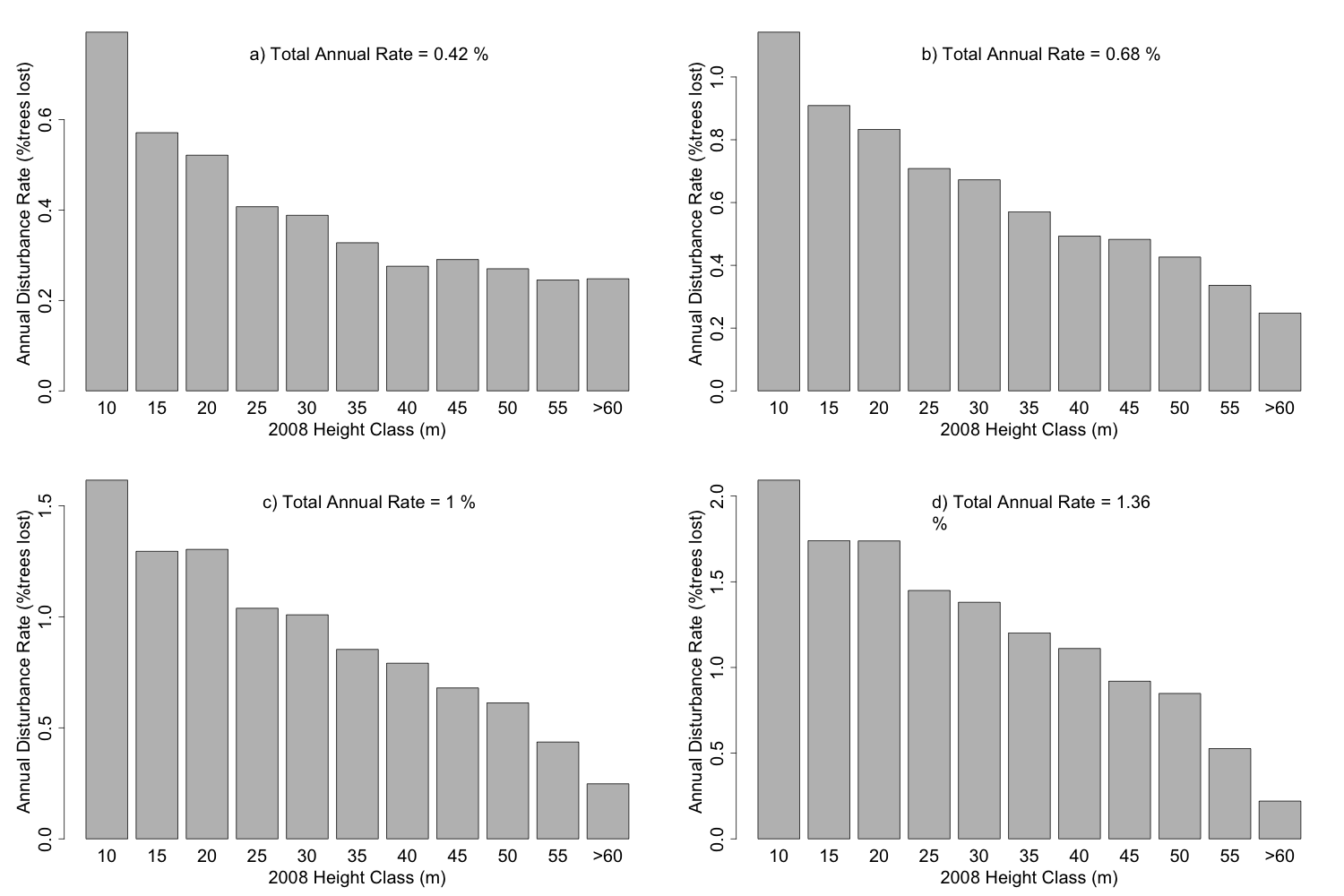


SI Fig 6. Is distance within 1* the radius, b) 2*, c) 3* and d) 4*


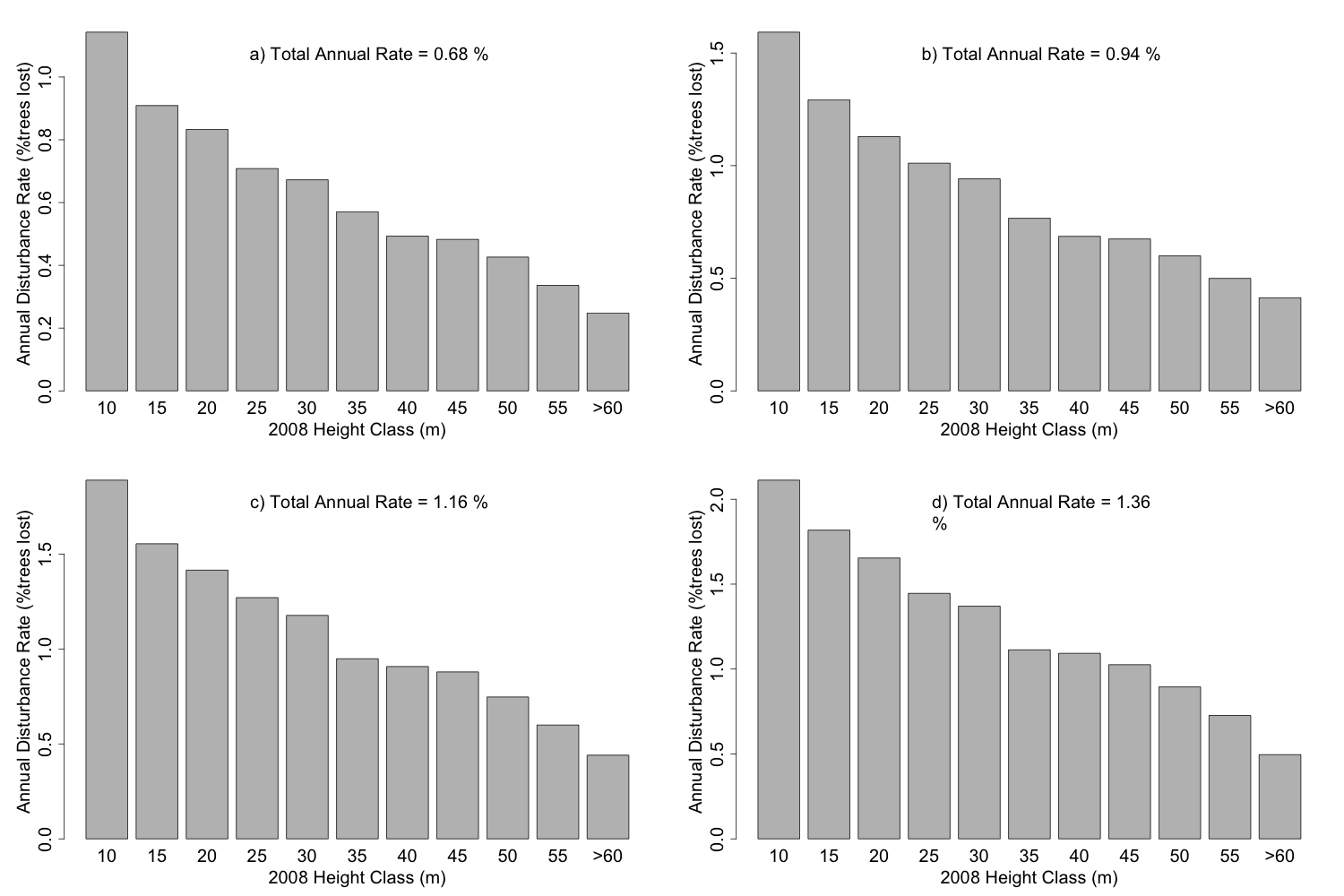


SI Fig 7. a) Within 2 m height b) within 3, c) within 4 and d) within 5.
